# A Case Series of Continuous Theta Burst Stimulation Treatment for the Supplementary Motor Area Twice a Day in Patients with Obsessive-Compulsive Disorder: A Real World TMS Registry Study in Japan

**DOI:** 10.3390/jpm13050875

**Published:** 2023-05-22

**Authors:** Yoshihiro Noda, Kyoshiro Fujii, Fumi Tokura, Shinichiro Nakajima, Ryosuke Kitahata

**Affiliations:** 1 Department of Neuropsychiatry, Keio University School of Medicine, Tokyo 160-8582, Japan; 2 Shinjuku-Yoyogi Mental Lab Clinic, Tokyo 151-0051, Japan

**Keywords:** continuous theta burst stimulation, obsessive-compulsive disorder, real-world research, supplementary motor area, transcranial magnetic stimulation, TMS registry study

## Abstract

Obsessive-compulsive disorder (OCD) is a psychiatric disorder characterized by patterns in which unwanted thoughts and fears are evoked as obsessions and furthermore, compulsive behaviors are provoked repeatedly, with a prevalence rate of 2% of the population. These obsessive-compulsive symptoms disrupt daily life and cause great distress to the individual. At present, OCD is treated with antidepressants, mainly selective serotonin reuptake inhibitors, and psychotherapy, including the exposure and response prevention method. However, these approaches may only show a certain level of efficacy, and approximately 50% of patients with OCD show treatment resistance. This situation has led to the research and development of neuromodulation therapies, including transcranial magnetic stimulation treatment, for OCD worldwide in recent years. In this case series, we retrospectively analyzed the TMS registry data of continuous theta burst stimulation (cTBS) therapy targeting the bilateral supplementary motor cortex for six patients with OCD whose obsessive-compulsive symptoms had not improved with pharmacotherapy. The results suggest that treatment with cTBS for the bilateral supplementary motor area may reduce obsessive-compulsive symptoms in patients with OCD, despite the limitations of an open-label preliminary case series. The present findings warrant further validation with a randomized, sham-controlled trial with a larger sample size in the future.

## 1. Introduction

Obsessive-compulsive disorder (OCD) is a brain disorder that affects approximately 2% of the population and causes significant distress and impairment in critical areas of functioning, including the patient’s social functioning and quality of life, consuming at least one hour a day, with symptoms of obsessive thoughts, compulsive behaviors, or both [1]. Obsessions are persistent, undesirable thoughts, or images that cause distress and anxiety, and compulsions are repetitive behaviors or mental rituals aimed at neutralizing distress and anxiety. First-line treatment for OCD is cognitive-behavioral therapy with exposure and response prevention and selective serotonin reuptake inhibitors. Even among patients who receive these evidence-based psychotherapies and pharmacotherapies, approximately 50% of patients are treatment-resistant to these therapies and persist with obsessive-compulsive symptoms [2]. In fact, the therapeutic effects of pharmacotherapy and psychotherapy are still unsatisfactory, with more than 60% of patients relapsing [2]. Given the ongoing and significant disease burden of OCD, it is imperative to establish novel and optimized treatment modalities. In this context, the potential of neuromodulation therapies, including transcranial magnetic stimulation (TMS), transcranial direct current stimulation (tDCS), and deep brain stimulation (DBS), have been investigated in recent years [3,4,5].

In fact, one type of TMS therapy, deep TMS, has already been approved by the U.S. FDA for treatment-resistant OCD in 2018 [6]; however, the approved protocol includes a separate symptom provocation to induce moderate obsessive-compulsive distress before each TMS session, which is a delicate, demanding, and uncomfortable procedure to work with. On the other hand, there are still various types of neuromodulatory treatments for OCD which have been under research and development and have not yet been optimized. Among them, TMS offers significant advantages over DBS that requires surgery because TMS is a safe, noninvasive treatment [7]. The two main types of TMS currently in widespread use are rTMS and theta burst stimulation (TBS). rTMS stimulates the cortex with repeated single pulses at a fixed frequency, while TBS involves the embedding of three 50 Hz high-frequency gamma stimulations bursting into a sustained 5 Hz theta rhythm, 200 ms apart [8]. Compared to rTMS, TBS is characterized by its similarity to physiological rhythms and is more likely to induce long-term potentiation at the synapse [9,10,11]. In addition, TBS includes continuous TBS (cTBS) and intermittent TBS (iTBS), the former having an inhibitory effect on the cerebral cortex and the latter activating the cortex [12,13].

Furthermore, recent developments in neuroimaging studies have gradually revealed the neural bases of OCD. In particular, the cortical-striatal-thalamocortical (CSTC) circuit is known as the pathological network of OCD, and its components, the prefrontal cortex (PFC) including orbitofrontal cortex (OFC) and medial PFC (mPFC), supplementary motor area (SMA), premotor cortex, striatum, globus pallidus, thalamus, and anterior cingulate cortex (ACC) have been shown to be overexcited in patients with OCD [14,15,16]. Among those brain regions, there are four main target areas accessible by TMS: dorsolateral PFC (DLPFC), mPFC, OFC, and SMA. In fact, there have been clinical studies for each of these brain regions [17,18,19,20,21]. However, the OFC is a difficult target site to stimulate from the viewpoint of tolerability because the stimulation site is close to the eyebrow. In addition, because the mPFC and ACC sites are located deep in the brain, it is necessary to use special coils such as deep TMS coils to achieve effective magnetic stimulation. Furthermore, while stimulating the DLPFC has been shown to be effective for depression and other psychiatric disorders, this site is not implicated directly in the core pathophysiology of OCD. Thus, given the limitations of the range that a regular TMS coil can stimulate, the tolerability issues, and the pathological basis of OCD, a recent meta-analysis by Rehn et al. suggests that the SMA site is the most reasonable and promising target for TMS therapy for OCD [22]. Furthermore, the findings in the pilot study by Mantovani et al., that functional connectivity between the bilateral SMA and the subcortical basal ganglia and thalamus in the CSTC circuit was significantly reduced in patients with OCD who responded to treatment with MRI-guided, individualized, twice-daily 1 Hz-rTMS to the bilateral SMA, also support this idea [23].

Another point is whether TMS treatment should be combined with exposure and response prevention. This is because it is not clear at this point whether TMS therapy can be more effective in the treatment of OCD if it is given in combination with exposure and response prevention. Moreover, since exposure and response prevention impose a certain level of burden on both the practitioner and the patient, if the procedure is not necessary from the standpoint of therapeutic effect, then there is no reason for it.

Therefore, in this case series, we performed a total of 30 sessions of cTBS treatment targeting bilateral SMA [24] without any exposure and response prevention and aimed to retrospectively compare the effectiveness of the treatment with the results of our recent case series of OCD treated with TMS combined with exposure and response prevention [25].

## 2. Materials and Methods

### 2.1. Case Series Setting

This case series was conducted as part of the Real World TMS Registry Study (TMS Database Registry Consortium Research Project in Japan: TReC-J) (jRCT1050210059) [26] of patients with OCD who visited the Shinjuku-Yoyogi Mental Lab Clinic in Tokyo between 1 April 2021 and 31 March 2022. The study included 6 outpatients with OCD who were refractory to medication and exhibited obsessive-compulsive symptoms at the moderate level or higher. In this case series, only patients with OCD who met the following eligibility criteria were included. In addition, with regard to concomitant medications, no changes in medication were made from several months prior to the introduction of TMS treatment until after the completion of TMS treatment.

### 2.2. Eligibility Criteria

The eligibility criteria for this case series are as follows:

(1) Eighteen years of age or older;
(2) Patients who met the Diagnostic and Statistical Manual of Mental Disorders, 5th edition (DSM-5) definition of the diagnosis of OCD by consultation with certified psychiatrists, including a standardized interview;
(3) Patients who have failed to achieve subjective improvement in obsessive-compulsive symptoms with pharmacotherapy, including antidepressants;
(4) Patients with no previous history of convulsive seizures;
(5) Patients with no other apparent contraindications to TMS therapy;
(6) Patients who had received a minimum of 30 sessions of TMS treatment and clinical assessment up to at least the end point.

### 2.3. Clinical Measures

Since we focused this case series on treatment for OCD symptoms, the severity of OCD symptoms was assessed using the Yale–Brown Obsessive Compulsive Scale (Y-BOCS) [27] as the primary outcome. Clinical assessments with Y-BOCS were conducted at three time points: at baseline before the start of TMS treatment, at interim points after a total of 15 sessions, and at the final time point after a total of 30 sessions. The percentage reduction in the Y-BOCS score was defined as the primary outcome, with a 35% or greater reduction in the Y-BOCS score defined as a “treatment response” and a reduction between 25% and 34% of the Y-BOCS score as a “partial response” [28].

### 2.4. TMS Treatment Protocol

In this case series, bilateral SMA was targeted for treatment, with 2 sessions of inhibitory cTBS intervention (approximately 4 min intervention and approximately 30 min rest between sessions) per visit. In this cTBS protocol, one session was defined as a set of cTBS to the right SMA (approximately 1 min with a total of 600 pulses) and cTBS to the left SMA (also approximately 1 min with a total of 600 pulses). Here, the reason for the approximately 30 min break was to maximize the neuroplastic changes induced by the cTBS intervention as much as possible, while at the same time reducing the risk of seizure induction by consecutive cTBS interventions as much as possible. A total of 30 sessions (15 visits total) of TMS treatment were performed on all patients. The frequency of TMS treatment visits was 3 to 5 visits per week (i.e., 6 to 10 weeks), depending on patient availability. Note that the original cTBS protocol consisted of a triplet stimulus of 50 Hz gamma rhythm in a train of 5 Hz theta rhythm, stimulated continuously for 40 s, with a total of 600 pulses of TMS administered [8,29]. Therefore, a total of 2400 pulses of cTBS were conducted per day in this protocol.

The resting motor threshold (RMT) was defined as the minimum stimulus intensity to cause muscle contraction in the right abductor pollicis brevis muscle at rest, 50% of the time, with a single pulse of TMS administered to the left primary motor cortex. The SMA site was set as a target based on previous studies [30,31], with the site located on the sagittal midline and anterior to the Cz site by 15% of the length of the nasion–inion distance. During the TMS treatment, the TMS coil was placed perpendicular to the sagittal plane, with the coil apex oriented to the right and the coil handle to the left for the right SMA, while the coil apex was oriented in the 180-degree opposite direction for the left SMA. Here, the rationale for this coil placement for the SMA in this protocol was based on the coil placement in the intervention study targeting dorsomedial PFC by Downar et al. [32,33,34,35]. Stimulation intensity was based on the RMT described above, and both the left and right SMA were stimulated at 120% RMT intensity. The Cool-B70 coil and MagPro R30 device (MagVenture, Inc., Farum, Denmark) were used for the TMS treatment. Medications were kept constant throughout the treatment period with no changes in content or dosage.

### 2.5. Statistical Analysis

IBM SPSS Statistics 26 (IBM, Chicago, IL, USA) was used for statistical analysis. This case series was a preliminary open-label observational study to evaluate the efficacy of a modified TMS regimen targeting the SMA sites in patients with OCD. Thus, we conducted a repeated measure analysis of variance (ANOVA) with time (baseline, interim, and end point) as a within-subject factor and paired *t*-tests on the longitudinal changes in the primary outcome, Y-BOCS scores, before and after the TMS treatment intervention, based on the assumption that this treatment regimen would improve obsessive-compulsive symptoms in patients with OCD to some extent. In addition, as subanalyses, the relationship between clinical epidemiological data (sex and medication status) and percentage changes in the Y-BOCS score was also examined. The significance level was set at 0.05 in this study.

## 3. Results

### 3.1. Overview of Each Case Included in This Case Series

Case 1: A 45-year-old male patient suffering from OCD symptoms for 22 years had previously tried three different antidepressants, all of which were ineffective, with only noticeable gastrointestinal side effects. At the time of introduction of TMS, he had not received any medication for several years. He had a Y-BOCS score of 19 prior to the start of TMS, which was a moderate level of OCD.

Case 2: A 48-year-old female patient suffering from OCD symptoms for 26 years had previously tried two different antidepressants, all of which were completely ineffective. At the time of TMS induction, she had not received any medication for 3 years. Prior to the start of TMS, her Y-BOCS score was 25, indicating a severe level of OCD.

Case 3: A 32-year-old male patient suffering from OCD symptoms for 12 years had previously tried two different antidepressants but was unable to take them due to strong side effects, especially nausea. At the time of TMS introduction, he had not received any medication for 11 years. Prior to the start of TMS, he had a Y-BOCS score of 16, which was a moderate level of OCD.

Case 4: A 62-year-old female patient suffering from OCD symptoms for 24 years had previously tried four different antidepressants with little improvement for her obsessive-compulsive symptoms. At the time of TMS initiation, she had not received any medication for 6 years. Prior to the start of TMS, she had a Y-BOCS score of 34, the most severe level of OCD.

Case 5: A 37-year-old male patient suffering from OCD symptoms for 8 years had previously tried three different antidepressants with limited improvement for obsessive-compulsive symptoms. At the time of TMS introduction, he was taking only 7.5 mg of mirtazapine for 3 years. Since the patient was sensitive to the side effect of drowsiness with mirtazapine, a minimal dose of mirtazapine was prescribed. Prior to the start of TMS, he had a mild level of OCD with a Y-BOCS score of 13.

Case 6: A 30-year-old male patient suffering from OCD symptoms for 4 years had previously tried two different antidepressants, but the improvement of his obsessive-compulsive symptoms was limited. At the time of TMS introduction, he had been taking 10 mg of escitalopram for approximately 2 years. Prior to the start of TMS, he had a Y-BOCS score of 24, indicating a severe level of OCD.

### 3.2. Clinicoepidemiological Characteristics of Patients with OCD in this Study

A total of six patients with OCD were included in this case series. These included four males and two females with a mean age (±S.D.) of 42.3 (±11.9) years. Detailed clinico-epidemiological data and medication information at baseline, prior to the start of TMS therapy, are summarized in Table 1. The mean stimulation intensity of TMS was 59.3 (±8.9)%, and all patients received up to a total of 30 sessions of OCD-TMS regimen at a stimulation intensity of 120% RMT. Of the six patients with OCD, four were not taking any psychiatric medications and the remaining two were taking small doses of antidepressants (see Table 1).

### 3.3. Changes in the Y-BOCS Score with the TMS Regimen

**Figure 1:**
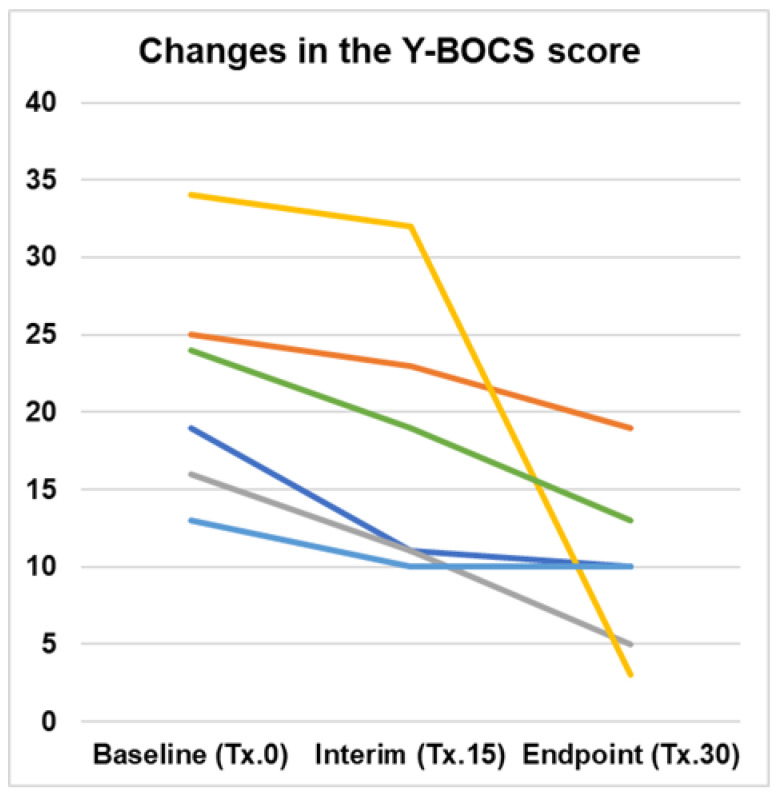
Longitudinal changes in the Y-BOCS score at the baseline, interim, and end point following cTBS treatment for the bilateral SMA in cases with OCD. Each colored line graph shows the change over time for each case. Each colored line graph shows the course of change in OCD symptoms for a different patient.

The Y-BOCS scores for the primary outcome improved from a mean (±S.D.) of 21.8 (±7.5) at baseline to 17.5 (±8.9) at the interim point after 15 treatment sessions and 10.0 (±5.7) at the end point after 30 treatment sessions in this case series. The ANOVA and post-hoc *t*-tests with Bonferroni correction showed a significant improvement from baseline to end point (F_2,15_ = 3.906, *p* = 0.043) but not between baseline and interim point. In terms of the mean percentage changes for the overall treatment, the results showed a 50.0 (±26.4)% improvement. The percentage of treatment response (≥35% improvement in the Y-BOCS score from the baseline) was 67% of the total. In this case series, none of the cases showed deterioration of the Y-BOCS scores due to TMS treatment. Longitudinal changes in the Y-BOCS score at the baseline, interim, and end point are graphically depicted in Figure 1.

### 3.4. Subanalyses of the Y-BOCS Score Changes

Differences in the Y-BOCS scores by sex and with or without concomitant medication were as follows. Male and female patients showed 46.3 (±18.7)% and 57.6 (±47.5)% improvement, respectively, with no significant difference in percentage changes between the two groups (t_4_ = 0.455, *p* = 0.673). In addition, patients who received no medication showed 57.8 (±28.8)% and patients with medication showed 34.5 (±16.1)% improvement, with no significant difference in percentage changes between the two groups (t_4_ = 1.031, *p* = 0.361). In addition, in this case series, there was no direct relationship between therapeutic effect on the Y-BOCS score and the severity of OCD or the length of the disease in each case.

### 3.5. Adverse Events Associated with the TMS Regimen Specific to the OCD Treatment

In this case series, no apparent adverse events associated with TMS treatment were observed. Note that there were no particular complaints of scalp pain associated with stimulation, which is often common with TMS treatment.

## 4. Discussion

### 4.1. Summary of the Results

In this case series, using the TMS registry data in Japan, six patients with OCD with mild to most severe severity on the Y-BOCS score were treated with bilateral SMA-targeted cTBS twice daily, for a total of 30 sessions. As a result, the Y-BOCS score improved by an average of 50 (±26.4)%, and the overall percentage of treatment response reached 67%. Moreover, no apparent adverse events were observed in all patients. Despite the limitations of the small number of cases and the preliminary open-label, observational study, this study showed that this TMS treatment regimen may be beneficial for OCD.

### 4.2. Neurophysiological Underpinnings of the SMA-cTBS Alleviating Obsessive-Compulsive Symptoms

The pathophysiology of OCD involves dysfunction of the CSTC circuit, which is responsible for motor execution, habit formation, and reward control, and in particular, overactivity of the CSTC circuit is thought to cause OCD symptoms, including obsessions and compulsive behaviors [14,15]. To date, target sites for TMS therapeutic intervention studies in OCD have ranged from brain regions indirectly involved in the pathogenesis of OCD (e.g., DLPFC, mPFC, and ACC) to brain regions more directly involved (e.g., pre-SMA/SMA and OFC) [19,20,36]. When the DLPFC, mPFC, and ACC are targeted for treatment, they may indirectly alleviate obsessive-compulsive symptoms by exerting proactive effects on depressive symptoms and cognitive decline associated with OCD through mechanisms similar to TMS treatment for depression, while when the pre-SMA/SMA and OFC are targeted as therapeutic targets, they may be a more direct and rational therapeutic approach to the pathophysiology of OCD, as they aim to normalize the pathological overactivity by directly neuromodulating a portion of the OCD circuitry. However, in the case of pre-SMA/SMA and OFC, findings from previous studies indicated that at least 20 sessions of TMS treatment are required to achieve a favorable therapeutic effect [21].

Previous TMS treatment studies targeting pre-SMA/SMA in OCD have shown symptom improvement in the range of 20% to 80% and have further found that more treatment sessions are associated with better treatment efficacy [37]. Another previous study comparable to our case series was that of Mantovani et al., in which they employed a protocol of low-frequency 1 Hz rTMS targeting bilateral SMA for a total of 7200 pulses twice daily at a 110% RMT stimulation intensity for 1 week in nine patients with medication-resistant OCD [23]. In their pilot study, they found a favorable improvement in OCD symptoms, assuming that the underlying treatment mechanism may be that the hyperexcited connectivity pattern between the bilateral SMAs and subcortical areas (especially the basal ganglia and thalamus) in the CSTC circuit was normalized by inhibitory, low-frequency rTMS, which in turn resulted in the improvement of OCD symptoms. The difference between our TMS protocol in this OCD case series and that of the pilot study by Mantovani et al. was that Mantovani et al. administered low-frequency 1 Hz rTMS to bilateral SMA with a total of 7200 pulses per day (approximately 2 h), whereas we employed cTBS protocol with a total of 2400 pulses per day (approximately 4 min). In addition, Mantovani et al. conducted personalized neuronavigation in their study, but we did not do that in our case series. Although there were some differences in the TMS intervention protocols between their study and our study, each study showed significant improvement in therapeutic efficacy for OCD symptoms, with no apparent adverse events. However, in light of the clinical outcomes described below for this case series, the method we used in this study of two sessions of cTBS per day targeting the SMA (with a total of 2400 pulses of intervention for 4 min with a 30 min break between sessions) without personalized neuronavigation seems to be a reasonable approach that imposes less burden on both the TMS providers and the patients. Moreover, in both studies, the mechanisms of action and therapeutic mechanisms assumed in the TMS intervention protocols for SMA were also the same.

In fact, in our case series, a total of 30 sessions of twice daily cTBS treatment for the bilateral SMA resulted in an overall average improvement rate by a Y-BOCS score of 50% (range of improvement: 23–91%), which is comparable to the treatment outcomes in the prior studies [30,31,38,39,40,41,42,43]. Furthermore, this TMS therapeutic regimen consists of a total of 30 sessions of cTBS treatment with two sessions of intervention (approximately 30 min between sessions) per day and three to five visits per week, which is a highly feasible and treatment-efficient protocol for patients, given that the treatment outcome is comparable to the results of previous studies.

### 4.3. Considerations on the Implication of Combining Exposure and Reaction Prevention Methods with TMS Treatment

In our recent case series of a different TMS protocol for 26 patients with OCD [25], we stimulated the medial prefrontal cortex as the therapeutic target with deep TMS using a butterfly coil at high frequency and combined this with an exposure and reaction prevention method before each session of rTMS, and we found that approximately 54% of patients responded to this combined treatment protocol. On the other hand, although the number of patients in this case series was small, the treatment response rate of this study was 67%, even without the combination of the exposure and response prevention method, indicating that the SMA-cTBS therapeutic intervention may be promising as a neuromodulation treatment for OCD, since it has the advantage of a short treatment time within a few minutes, as well as an overall intervention period of 3 to 5 weeks.

In addition, when the exposure and response prevention method is used in combination with TMS treatment, the practitioner’s procedure becomes more complicated and the psychological burden on the patient increases. Thus, it is reasonable if the exposure and response prevention method is not necessary to be used in combination with TMS treatment, as this will help to reduce the physical and psychological burden on the practitioner and patient. From another perspective, since the implementation of the exposure and response prevention method in the clinical setting itself has a certain level of limitation, it is unrealistic for patients and may even be less effective as a treatment method, as they are merely simulating a feeling that is qualitatively and quantitatively different from their actual distress. Therefore, a more realistic approach to address this unmet medical need in the future may be to develop and establish neuromodulation methods that directly approach the neurobiological basis of OCD in a simpler manner.

### 4.4. Limitation and Future Direction

This study has several limitations. First, since the number of patients in this case series is only six, the results of this analysis are still preliminary. Second, since the present case series was a retrospective observational study using a subset of the Japanese TMS registry data, more rigorous validation with a prospective randomized controlled trial (RCT) design is needed in the future. Third, since MRI-guided neuronavigation was not used in this case series, it may be necessary to refine the stimulation target site in the future to improve the therapeutic effect. Fourth, the subanalyses in this case series on the effects of sex and concomitant medication on TMS treatment should be considered preliminary results for reference, only due to the tiny sample size, and should await validation with large-scale data in the future.

Therefore, to overcome the above limitations, as a future direction, it is necessary to further increase the sample size and confirm the reproducibility and generalizability of the results. Furthermore, the follow-up assessment of obsessive-compulsive symptoms by Y-BOCS after the completion of TMS treatment, especially regular follow-up assessments of symptom relapse/recurrence and preventive interventions against them, should also be considered in the future. In addition, when conducting a prospective RCT, it is necessary to incorporate biological measures such as MRI and EEG to elucidate the therapeutic mechanism of TMS treatment for OCD.

## 5. Conclusions

This observational study of SMA-cTBS treatment in patients with OCD, using the real-world TMS therapy registry data in Japan, showed that cTBS treatment may have a positive impact on the mitigation of obsessive-compulsive symptoms for patients with OCD. Future large-scale TMS treatment studies with an RCT design should be conducted to establish an effective and safe TMS treatment protocol to alleviate obsessive-compulsive symptoms in patients with OCD.

## Figures and Tables

**Table 1:** Clinico-demographic information.

Characteristics	
Number of individuals; age (mean ± S.D.) [years]	n = 6; 42.3 (±11.9)
Males; age (mean ± S.D.) [years]	n = 4; 36.0 (±6.7)
Females; age (mean ± S.D.) [years]	n = 2; 55.0 (±9.9)
Duration of an illness [years]	16.0 (±9.2)
Baseline Y-BOCS score (mean ± S.D.)	21.8 (±7.5)
Resting motor threshold (RMT) [%]	49.3 (±7.6)
Stimulus intensity for the bilateral SMA (%MSO)	59.3 (±8.9) (120% RMT)
**Medication Status**	
Four patients were off medication.	None.
Two patients were on antidepressants.	One patient was taking mirtazapine 7.5 mg and another escitalopram 10 mg.

S.D.: standard deviation; SMA: supplementary motor area; RMT: resting motor threshold; MSO: maximum stimulator output.

## Data Availability

All clinical data are available upon reasonable request to the corresponding author.
